# The Effect of Prophylactic Adnexectomy on the Quality of Life and Psychosocial Functioning of Women with the *BRCA1/BRCA2* Mutations

**DOI:** 10.3390/ijerph16244995

**Published:** 2019-12-09

**Authors:** Marta Stanisz, Mariusz Panczyk, Rafał Kurzawa, Elżbieta Grochans

**Affiliations:** 1 Department of Gynecology and Reproductive Health, Pomeranian Medical University in Szczecin, 71-210 Szczecin, Poland; martastanisz@gmail.com (M.S.); kurzawa@pum.edu.pl (R.K.); 2 Department of Education and Research in Health Sciences, Medical University of Warsaw, 02-091 Warsaw, Poland; mariusz.panczyk@wum.edu.pl; 3 Center of Gynecology and Treatmemt for Infertility “Vitrolive”, al. Wojska Polskiego 103, 70-483 Szczecin, Poland; 4 Department of Nursing, Pomeranian Medical University in Szczecin; ul. Żołnierska 48, 71-210 Szczecin, Poland

**Keywords:** *BRCA1*, *BRCA2*, risk-reducing salpingo-oophorectomy, quality of life, psychosocial functioning, ovarian cancer

## Abstract

The main purpose of this study was to analyze the effect of risk-reducing salpingo-oophorectomy (RRSO) on the quality of life (QoL) and psychosocial functioning of patients with the *BRCA1/BRCA2* mutations. This survey-based study was conducted using the Blatt-Kupperman Index, the Women’s Health Questionnaire, the Perceived Stress Scale, the State-Trait Anxiety Inventory, the Beck Depression Inventory-II, and the authors’ questionnaire. All calculations were done using Statistica 13.3. The QoL after RRSO was statistically significantly lower in most domains compared with the state before surgery. The greatest decline in the QoL was observed in the vasomotor symptoms domain (*d* = 0.953) and the smallest in the memory/concentration domain (*d* = 0.167). We observed a statistically significant decrease in the level of anxiety as a state (*d* = 0.381), as well as a statistically significant increase in the severity of climacteric symptoms (*d* = 0.315) and depressive symptoms (*d* = 0.125). Prophylactic surgeries of the reproductive organs have a negative effect on the QoL and psychosocial functioning of women with the *BRCA1/2* mutations, as they increase the severity of depressive and climacteric symptoms. At the same time, these surgeries reduce anxiety as a state, which may be associated with the elimination of cancerophobia.

## 1. Introduction

*BRCA1* and *BRCA2* are human suppressor genes responsible for the control and regulation of the cellular cycle and the DNA repair mechanisms [1,2,3]. Mutations in these genes promote abnormal cell division and consequently contribute to oncological diseases [4,5]. Estimates show that from 1 out of 800 people to 1 out of 300 people in the general population is a carrier of a mutation in one of these genes [6]. Genetic testing has been performed worldwide since the mid-1990s in order to prevent oncological diseases in carriers of defective genes. Women with the *BRCA1* and *BRCA2* mutations are predisposed to malignant tumors located mainly in the ovaries, fallopian tubes, breast, and peritoneum, but also in other organs.

The lifetime risk of ovarian cancer in the general population is 1.5%–2%, and the risk of breast cancer is 8%–10%. According to the estimates, about 10% of all ovarian cancer and 3%–5% of breast cancer cases are caused by mutations in the *BRCA1* or *BRCA2* genes [7,8,9]. In the group of carriers of the *BRCA1* mutation, the lifetime risks of developing ovarian and fallopian tube cancers oscillate around 15% and 54%, respectively [10], and the risk of breast cancer ranges from 60% to 80% [10,11]. For carriers of the *BRCA2* mutation, the risk of ovarian cancer is 11%–16.5%, and the risk of breast cancer is 45%–55% [12]. Moreover, women with this mutation are more likely to develop cancer in the second breast and to have a recurrence after therapy. Ovarian cancer associated with the *BRCA1/BRCA2* mutations usually occurs about ten years earlier (usually in the fourth decade of a woman’s life) and is characterized by a substantially higher clinical stage compared with the general population (stage III and IV disease according to the International Federation of Gynecology and Obstetrics (FIGO) classification system) [13]. Moreover, the risk of developing cancer in this group of women increases on average by 10% after each subsequent decade of life.

Despite the increasing availability of modern diagnostic and therapeutic methods, ovarian cancer remains a challenge for oncological gynecology, mainly due to late diagnosis and consequent poor prognosis [14]. Unfortunately, screening for ovarian cancer as a part of ovarian cancer prevention is not precise enough to detect 100% of neoplastic lesions. Thus, it can be concluded that the mortality rate for this cancer cannot be reduced only by screening and chemoprevention [15].

Surgical intervention is an alternative, although radical, method of prevention applied in carriers of the *BRCA1/BRCA2* mutations. Currently, it is regarded as the only effective method to prevent the development of cancer in the ovaries, fallopian tubes, and breast [16]. Prophylactic surgeries—considered as a “golden standard”—involve salpingo-oophorectomy, sometimes accompanied by the removal of the body of the uterus or hysterectomy, depending on individual indications.

The health and psychological consequences of risk-reducing salpingo-oophorectomy (RRSO) are in opposition to its potential antitumor effect. The majority of patients who decide to undergo prophylactic adnexectomy are aware that its benefits outweigh its disadvantages. Undoubtedly, such a surgery decreases anxiety associated with the risk of developing cancer. Unfortunately, it can also negatively affect the physical and mental state of women, as well as their ability to fulfill previous roles in society [17,18], resulting in lower quality of life (QoL) and worse psychosocial functioning.

### Aim of the Study

The main purpose of this study was to analyze the effect of prophylactic surgery of the reproductive organs on the QoL and selected aspects of psychosocial functioning of patients with the *BRCA1/BRCA2* mutations. A specific objective was to analyze the relationship of the level of stress, depressive symptoms, anxiety as a trait, and anxiety as a state with the QoL before and after RRSO.

## 2. Materials and Methods

### 2.1. Participants

The study involved 62 patients with the *BRCA1* (95.1%) and *BRCA2* (4.9%) mutations referred to the Clinic of Surgical Gynecology and Gynecologic Oncology for Women and Girls, Independent Public Clinical Hospital No. 2, Pomeranian Medical University in Szczecin (western Poland) to undergo bilateral prophylactic adnexectomy (75.4%), prophylactic removal of the uterus and adnexa (23.0%), or prophylactic total laparoscopic hysterectomy accompanied by adnexectomy (1.6%). The mean age was 44.7 ± 9.35; the median (Me) was 41 years.

The criteria for inclusion in the study were a documented mutation in the *BRCA1* or *BRCA2* gene, a normal pap smear result, documented results of the CA125 and HE4 analysis falling within normal limits, written informed consent of the patient to take part in the study, and completing the set of questionnaires given by the researchers.

The criteria for exclusion from the study were not meeting all the inclusion criteria, a lack of written consent for participation in the study, or suspected ovarian cancer or a lesion in the adnexa area requiring further diagnosis or surgical intervention other than prophylactic surgery.

The majority of the women undergoing surgery had secondary (52.4%) and higher (44.3%) education. The least numerous patients were those with only primary education (3.3%). Most respondents (77.0%) were married, 11.5% were divorced, 8.2% were single, and 3.3% were widows. At the moment of deciding to undergo prophylactic surgery of the reproductive organs, 62.3% of the women were before menopause, and 37.7% were after menopause. Numerous women (57.4%) were also those in whom breast cancer had been diagnosed and treated prior to prophylactic surgery. In one patient, histopathological examination of the material obtained during prophylactic adnexectomy revealed ovarian cancer. The diagnosis necessitated further surgical and oncological therapy.

### 2.2. Course of the Study

The study was conducted at two stages: prior to prophylactic surgery of the reproductive organs—during hospitalization via personal contact with a patient—and after prophylactic surgery of the reproductive organs—after about one year via e-mail, mail, phone, or personal contact, depending on the patient’s preference and place of stay.

The average time from the prophylactic surgery of the reproductive organs (the time between the first and second stages of the study) was 353 days. The respondents qualified for the study were informed about its purpose, course, and the fact that it had no impact on therapy and further medical management. Each time, oral and written consent to participate in the experiment were obtained from the women.

Before starting work on the project, we obtained the approval of the Bioethical Commission of the Pomeranian Medical University in Szczecin (Poland) (approval no. KB-0012/117/15), as well as all necessary licenses and permits to use standardized research instruments.

### 2.3. Research Instruments

The study was based on a survey and analysis of medical documentation, using the following research instruments:

1. **The Blatt-Kupperman Index** consists of 11 statements concerning the severity of menopausal symptoms (such as vasomotor symptoms, paraesthesia, insomnia, nervousness, melancholia, vertigo, fatigue, arthralgia, headache, palpitations, formication) rated by respondents on a four-point scale. The following norms were accepted: a score of 0–15, no climacteric symptoms; 15–20, slight climacteric symptoms; 20–35, moderate climacteric symptoms; and more than 35, severe climacteric symptoms [19].
2. **The Polish adaptation of the Women’s Health Questionnaire (WHQ)** was developed by Myra Hunter in the 1980s. It serves to measure self-reported somatic and psychological symptoms faced by menopausal women. This instrument consists of 36 questions, responses to which are rated on a four-point scale. The WHQ enables a reliable assessment of nine aspects of emotional and physical health, namely, depressed mood (DEP), somatic symptoms (SOM), memory/concentration (MEM), vasomotor symptoms (VAS), anxiety/fears (ANX), sexual behavior (SEX), sleep problems (SLE), menstrual symptoms (MEN), and attractiveness (ATT). The interpretation of the raw data is based on the standards for various age brackets proposed by the author of the instrument [20].
3. **The Perceived Stress Scale (PSS-10)** is a psychological instrument for measuring the degree to which situations in one’s life are appraised as stressful. The PSS consists of 10 questions asking about feelings and thoughts during the last four weeks. Responses are weighed on a five-point scale. The raw results are obtained through summing up individual scores after transforming them according to the key. The results converted into standardized sten norms are interpreted as follows: 1–4, low score; 5–6, average score; 7–10, high score [21].
4. **The State-Trait Anxiety Inventory (STAI)** consists of two independent parts. The first part, STAI (X-1), measures the level of anxiety understood as the transitory and situationally conditioned status of an individual (anxiety as a state). The second part, STAI (X-2), concerns anxiety understood as a relatively permanent personality trait (anxiety as a trait). Each part includes a set of 20 statements. Respondents take a stance on each statement, choosing one of four possible answers. The scores are calculated through summing up points obtained for separate answers. Raw data are converted into standardized sten results for gender and age. The sten scale is interpreted as follows: 1–4, low levels of anxiety as a trait and as a state; 5–6, average levels of anxiety as a trait and a state; 7–10, high levels of anxiety as a trait and a state [22].
5. **The Beck Depression Inventory-II (BDI-II)** is a 21-item self-report measure of depressive symptoms. Responses are rated on a four-point scale (0–3), then the scores are added up to give a final score. The interpretation of the results is as follows: 0–13 points, no depression or minimal depressive symptoms; 14–19 points, mild depression; 20–28 points, moderate depression; and 29–63 points, severe depression [23].
6. **The authors’ questionnaire** consists of two versions to be used before and after prophylactic surgery, designed to collect sociodemographic and medical data and to assess the decision to undergo surgery and opinions about psychological care received before and after surgery.

### 2.4. Statistical Analysis

The variables were characterized using the parameters of descriptive statistics, the selection of which was determined by the type of the measurement scale. The “before RRSO” and “after RRSO” results were compared using Student’s *t*-test for dependent samples. The effect size for the difference between the samples was calculated using Cohen’s *d* coefficient [24]. The difference was regarded as small if (*d*: 0.10–0.30), average if (*d*: 0.31–0.50), and large if (*d* > 0.50) [25]. The relationship between the patients’ QoL and the levels of selected variables, such as climacteric symptoms, stress, and anxiety, was calculated using Spearman’s correlation coefficient (rho) [26]. The influence of sociodemographic and medical data on the women’s QoL and psychosocial functioning was assessed by the Mann–Whitney *U* test. The effect size for the difference was calculated using the eta squared (η^2^) coefficient [24]. The difference was regarded as small if (η^2^: 0.010–0.039), average if (η^2^: 0.040–0.110), and large if (η^2^ > 0.110) [25].

All calculations were done using Statistica 13.3 (Tibco^®^, Inc., USA, 2017). The level of statistical significance assumed a priori for the null hypothesis was 0.05.

## 3. Results

The analysis demonstrated statistically significant differences in the women’s QoL before and after prophylactic surgery of the reproductive organs. The QoL after prophylactic surgery of the reproductive organs was statistically significantly lower in the domains of somatic symptoms (SOM), memory/concentration (MEM), vasomotor symptoms (VAS), sexual behavior (SEX), and sleep problems (SLE) compared with the state before surgery. The greatest decline in the QoL was observed in the vasomotor symptoms (VAS) domain (*d* = 0.953) and the smallest in the memory/concentration (MEM) domain (*d* = 0.167). The effect size was only calculated for statistically significant differences (Table 1).

The mean sten score for anxiety as a state (STAI X-1) before and after surgery was average. The mean level of anxiety as a trait (STAI X-2), determined only once (before surgery), was 4.4 ± 2.2, which oscillated between a low and an average result on the sten scale. Analysis of the severity of climacteric symptoms revealed significant differences in the mean levels in the study sample. The mean levels of stress and depressive symptoms before adnexectomy were lower than those after this surgery.

Statistically significant changes were demonstrated in three aspects of the women’s psychosocial functioning before and after prophylactic surgery of the reproductive organs. We observed a statistically significant decrease in the level of anxiety as a state (STAI X-1) (*d* = 0.381), as well as a statistically significant increase in the severity of climacteric symptoms (*d* = 0.315) and depressive symptoms (*d* = 0.125). There was no statistically significant difference in the level of stress. The size of the difference for anxiety as a state (STAI X-1) and for the severity of climacteric symptoms was similar, while for depressive symptoms it was noticeably smaller (Table 2).

Analysis demonstrated a statistically significant negative correlation between the level of anxiety as a state (STAI X-1) and the women’s QoL in all domains, except for vasomotor symptoms (VAS) and menstrual symptoms (MEN), before RRSO. The strongest correlations were observed in the domains of depressed mood (DEP) and anxiety/fears (ANX). A statistically significant negative correlation between the level of anxiety as a state (STAI X-1) and the QoL was also demonstrated after removal of the reproductive organ, but only in the domains of depressed mood (DEP), anxiety/fears (ANX), sleep problems (SLE), and attractiveness (ATT) (Table 3).

Analysis demonstrated a statistically significant negative correlation between the level of anxiety as a trait (STAI X-2) and the women’s QoL before prophylactic surgery of the reproductive organs in all domains, except for vasomotor symptoms (VAS). The strongest correlations were noted in the domains of depressed mood (DEP), anxiety/fears (ANX), and attractiveness (ATT). There was also a statistically significant negative correlation between the level of anxiety as a trait (STAI X-2) and the women’s QoL after RRSO in all domains, except for vasomotor symptoms (VAS), sexual behavior (SEX), and menstrual symptoms (MEN). The strongest correlations were observed in the domains of depressed mood (DEP), anxiety/fears (ANX), and sleep problems (SLE) (Table 4).

The women with higher stress levels had lower QoL (negative correlation) in all domains, apart from vasomotor symptoms (VAS), both before and after RRSO. The strongest correlations were found in the domains of depressed mood (DEP), anxiety/fears (ANX), memory/concentration (MEM), sleep problems (SLE), and attractiveness (ATT) before surgery. After RRSO, on the other hand, the strongest correlations were observed in the domains of depressed mood (DEP), anxiety/fears (ANX), sleep problems (SLE), and memory/concentration (MEM) (Table 5).

The women with more severe depressive symptoms before RRSO had lower QoL (negative correlation) in all domains, except for vasomotor symptoms (VAS). The strongest correlations were noted in the domains of depressed mood (DEP), anxiety/fears (ANX), memory/concentration (MEM), attractiveness (ATT), and sleep problems (SLE). The women with more severe depressive symptoms had lower QoL in all domains, except for vasomotor symptoms (VAS), and menstrual symptoms (MEN) also after prophylactic surgery of the reproductive organs. The strongest correlations after RRSO were demonstrated in the domains of depressed mood (DEP), anxiety/fears (ANX), sleep problems (SLE), and memory/concentration (MEM) (Table 6).

## 4. Discussion

The influence of preventive surgery on the health, QoL, and psychosocial functioning of women with the *BRCA1/BRCA2* mutations has been continually discussed due to the numerous and varied consequences of such interventions. To date, prophylactic adnexectomy has often been performed during hysterectomy. Thus, the ovaries and fallopian tubes are those healthy organs that are most often removed without a medical indication [27].

Nevertheless, in view of the lack of other effective methods to prevent and treat ovarian cancer, prophylactic adnexectomy is beneficial, especially for genetically predisposed women, as it reduces the risk of cancer by 80% to 96% [28,29]. The percentage of patients after surgery whose susceptibility to ovarian cancer is confirmed by histopathological examination ranges from 2.6% to 6%, which corresponds with the results of our investigation, clinical observation, and reports from other authors [30,31,32].

The majority of the sparse studies on the QoL of women with the *BRCA1/BRCA2* mutations have been performed after prophylactic adnexectomy, and the results were compared to those obtained for the general population and patients who underwent screening. Fry et al. debated about a significant decline in the QoL after RRSO as early as in 2001, and the findings published in the subsequent years were also divergent [33]. Elit et al. (2001) demonstrated a lack of significant differences between women after surgery and the general public (*N* = 40) [34]. Similar results were obtained by Madalinska et al. (2006), and by Robson et al. (2003), who analyzed women subjected to RRSO two years before the study [35,36]. Our analysis concerned changes in the QoL and psychosocial functioning in the same study sample before and after prophylactic surgery. Analogous research carried out by Finch et al. (2013) in 2003–2008 (*N* = 96), showing no significant differences between the functioning of women after RRSO and the functioning of the general population, demonstrated the negative impact of this surgery on the physical and mental health of nearly one-fifth of all women subjected to this intervention [37]. Johansen et al. (2016), who compared the functioning of the control group (*n* = 1228) and that of women subjected to RRSO between 1978 and 2005 (*n* = 294), provided evidence that the patients after prophylactic surgery of the reproductive organs had significantly higher self-reported overall QoL, especially in the domains of physical functioning and playing roles [38]. However, in our study, the results for most of the nine domains assessed by the Women′s Health Questionnaire (WHQ) were decreased when compared with the state before surgery. Considerable changes in the QoL levels were related to the severity of vasomotor symptoms (VAS) and somatic symptoms (SOM), which corresponds with other authors’ findings, showing that patients after RRSO have considerably more severe climacteric symptoms, even though their overall health results are better than those of women deciding to have screening [34,35,39].

Similarly to other researchers, we observed substantial worsening of sleep problems [40] associated with a low level of estradiol [41]. Thus, patients after prophylactic surgeries of the reproductive organs may be at a higher risk of developing sleep disorders and may experience them with greater severity. This, however, requires further analysis and rational management on a clinical level.

Not only does adnexectomy entail the loss of fertility but it also causes a drastic decline in the level of sex hormones, determining sexual behaviors. In our investigation, we observed the adverse effect of RRSO on the sexual sphere, reflected by significantly lower scores for the sexual behavior (SEX) domain. These results have been confirmed by numerous studies [37,38]. As indicated by Fang et al. (2009), women in this group are noticeably less sexually active [42]. Tucker et al. (2016) pointed out a high incidence of sexual dysfunctions and sexual desire disorders among women after RRSO (*N* = 119) compared with the general population. At the same time, they emphasized that only a small percentage of patients are dissatisfied with their sexual life [43], which can be associated with their low expectations for intimate relationships, their partners, and themselves. One of the predictive factors for sexual activity of patients after RRSO is satisfaction with the relationship with the partner, which protects women against sexual passivity, even if they face complaints typical of the climacteric period [44]. On account of that, research on women’s sexuality and satisfaction with sexual life should also involve their partners, which would allow drawing general conclusions. The literature provides evidence that prophylactic surgeries of the reproductive organs that have an effect on the intimate area constitute a serious clinical problem. It seems reasonable to open centers offering counselling on sexual issues. Bonadies (2011) indicates that over half of patients declare the need for such services [45].

In our investigation, a substantial decrease in the QoL level was also observed in the memory/concentration (MEM) domain. Since the majority of patients undergoing RRSO are young women, impaired cognitive function can make their functioning considerably more difficult. Moreover, these women are at a higher risk of developing Alzheimer’s disease [46]. The long-term effects of RRSO on cognitive function and the nervous system are not known, but Zeydan et al. (2018) suggest that a sudden change in hormonal status associated with adnexectomy in premenopausal women can lead to abnormalities in the structure of the temporal lobe and, consequently, to cognitive disorders later in life [47].

Aside from QoL, another essential factor contributing to the functioning of these patients is anxiety, understood mainly as the fear of developing cancer. Our comparison of the data collected before and after RRSO showed a significant decrease in the severity of anxiety as a state (STAI X-1) in the whole study sample. The results reported by Lorenz et al. (2014) and other authors correspond with ours [44,48]. Finch et al. (2013) noticed a significantly lower level of anxiety in women subjected to RRSO before menopause compared with those operated upon after menopause [37]. Thus, reduced anxiety observed after RRSO confirms that this surgery has a positive impact on women’s mental state. Thus, prophylactic surgery of the reproductive organs not only decreases the mortality rate among patients with the *BRCA1/BRCA2* mutations but also has a positive emotional effect, as it eliminates the patient’s constant anxiety about their own future, health, and, in particular, life.

A key component of psychosocial functioning is also depression. We noted a considerable increase in the severity of depressive symptoms in the carriers of the *BRCA1/BRCA2* mutations after RRSO. Finch et al. (2013), however, claim that this aspect of functioning does not change after prophylactic surgeries, which is in line with the previous reports of other authors [35,42]. Nevertheless, many studies indicate that women during menopause are prone to depression, which involves the necessity of screening for this health problem [49]. Unfortunately, the psychological state of a patient, which is extremely important for the overall health assessment, is often ignored. Our findings stand in contradiction to a few reports by other authors. Thus, they are a vital element of a discourse, and the one that has not so far been analyzed in detail. Therefore, it seems necessary to undertake further research on the psychosocial effects of RRSO among women with the *BRCA1/2* mutations.

## 5. Conclusions

Prophylactic surgeries of the reproductive organs have a negative effect on the QoL and psychosocial functioning of women with the *BRCA1/2* mutations, as they increase the severity of depressive and climacteric symptoms. At the same time, these surgeries reduce anxiety as a state, which may be associated with the elimination of cancerophobia. Individual analysis of factors contributing to the QoL and psychosocial functioning is crucial for taking corrective actions.

Prior to giving informed consent to undergo prophylactic surgery of the reproductive organs, patients should be informed about not only the advantages of adnexectomy but also its short-term and long-term negative consequences, as well as the possibilities of their elimination.

Further research should be carried out to systematize the knowledge of the short-term and long-term consequences of prophylactic surgeries of the reproductive organs, performed in carriers of the *BRCA1/2* mutations, with regard to the health, psychosocial, and financial consequences of RRSO. A key problem is also the lack of guidelines on postoperative management of patients with the *BRCA1/2* mutations. Therefore, it is necessary to develop universal recommendations on preventive care, patient self-care education, and environmental adaptation after RRSO.

### Strengths and Limitations

Summing up, we should mention several factors that hindered analysis of the results. The most important of them was the size of the study sample (*N* = 61), although it was satisfactory in the context of the period when the study was carried out (January 2016–July 2018), the high questionnaire return rate achieved by an individual approach to the patients, and the sizes of the study samples described in other publications. In the majority of the available studies, particular stages of the research were carried out with long time intervals (sometimes over a dozen years) between RRSO and analysis of its impact on QoL, which may have had an effect on the results obtained, mainly because of dynamic changes in the quality and range of medical care. Furthermore, interpretation of the results is complicated due to the different methods applied. It would be beneficial to carry out multicenter research in order to systematize the often-contradictory findings. Nonetheless, a number of aspects presented and discussed in our study provide a basis for improving the care of patients with the *BRCA1/BRCA2* mutations, both before and after prophylactic surgeries of the reproductive organs.

## Figures and Tables

**Table 1:** The quality of life (QoL) of women with the *BRCA1/BRCA2* mutations before and after prophylactic surgery of the reproductive organs (*N* = 61).

QoL Domain	before RRSO			after RRSO			t	*p* *	Effect Size **
	M ± SD	Min–Max	CV [%]	M ± SD	Min–Max	CV [%]			
Depressed mood (DEP)	0.80 ± 0.26	0.14–1	32.2	0.77 ± 0.26	0.14–1	33.3	1.671	0.100	-
Somatic symptoms (SOM)	0.70 ± 0.25	0.14–1	35.9	0.61 ± 0.25	0.14–1	40.0	3.925	0.000	0.360
Memory/concentration (MEM)	0.70 ± 0.36	0–1	50.5	0.64 ± 0.36	0–1	56.6	2.168	0.034	0.167
Vasomotor symptoms (VAS)	0.66 ± 0.43	0–1	64.1	0.25 ± 0.32	0–1	127.6	7.743	0.000	0.953
Anxiety/fears (ANX)	0.82 ± 0.23	0.25–1	27.9	0.79 ± 0.26	0–1	32.3	1.803	0.076	-
Sexual behavior (SEX)	0.58 ± 0.36	0–1	61.4	0.48 ± 0.36	0–1	74.6	3.751	0.000	0.278
Sleep problems (SLE)	0.71 ± 0.32	0–1	45.8	0.59 ± 0.35	0–1	59.2	3.944	0.000	0.375
Menstrual symptoms (MEN)	0.78 ± 0.24	0.25–1	31.4	0.79 ± 0.24	0–1	30.8	−0.574	0.568	-
Attractiveness (ATT)	0.59 ± 0.35	0–1	58.9	0.60 ± 0.34	0–1	56.7	−0.299	0.766	-

RRSO, risk-reducing salpingo-oophorectomy; M ± SD, mean and standard deviation; Min–Max, minimum and maximum; CV, coefficient of variation; t, testing statistics. * Student’s *t*-test for dependent groups (repeated measurement), ** Cohen’s *d* coefficient.

**Table 2:** Psychosocial functioning of women with the *BRCA1/BRCA2* mutations before and after prophylactic surgery of the reproductive organs (*N* = 61).

Parameters of Psychosocial Functioning	before RRSO			after RRSO			t	*p* *	Effect Size **
	M ± SD	Min–Max	CV [%]	M ± SD	Min–Max	CV [%]			
Anxiety as a state (STAI X-1)	5.9 ± 2.10	1–10	35.3	5.1 ± 1.73	1–9	33.9	4.273	0.000	0.381
Anxiety as a trait (STAI X-2)	4.4 ± 2.20	1–10	49.6	-	-	-	-	-	-
Climacteric symptoms as determined by the Blatt-Kupperman Index	28.7 ± 24.16	0–104	84.1	36.3 ± 22.10	4–108	60.9	−6.561	0.000	0.315
Stress as determined by the PSS-10	5.5 ± 1.91	2–9	34.6	5.7 ± 1.85	2–10	32.7	−0.782	0.437	-
Depressive symptoms as determined by the BDI	8.2 ± 8.8	0–43	107.8	9.3 ± 8.70	0–43	93.1	−2.471	0.016	0.125

RRSO, risk-reducing salpingo-oophorectomy; M ± SD, mean and standard deviation; Min–Max, minimum and maximum; CV, coefficient of variation; t, testing statistics. * Student’s *t*-test for dependent groups (repeated measurement), ** Cohen’s *d* coefficient. STAI X-1—State-Trait Anxiety Inventory X-1. STAI X-2—State-Trait Anxiety Inventory X-2. PSS—Perceived Stress Scale. BDI—Beck Depression Inventory.

**Table 3:** The level of anxiety as a state as determined by the STAI X-1 and the QoL of women with the *BRCA1/BRCA2* mutations before and after prophylactic surgery of the reproductive organs (*N* = 61).

Level of Anxiety as a State According to STAI X-1	rho	*p*	rho	*p*
	before RRSO		after RRSO	
Depressed mood (DEP)	−0.61	0.000	−0.43	0.000
Somatic symptoms (SOM)	−0.45	0.000	−0.18	0.165
Memory/concentration (MEM)	−0.43	0.001	−0.18	0.156
Vasomotor symptoms (VAS)	−0.06	0.668	0.01	0.959
Anxiety/fears (ANX)	−0.60	0.000	−0.36	0.004
Sexual behavior (SEX)	−0.30	0.020	−0.01	0.938
Sleep problems (SLE)	−0.45	0.000	−0.33	0.009
Menstrual symptoms (MEN)	−0.19	0.151	−0.15	0.247
Attractiveness (ATT)	−0.49	0.000	−0.38	0.003

RRSO, risk-reducing salpingo-oophorectomy; rho, Spearman’s correlation coefficient; *p*, level of significance.

**Table 4:** The level of anxiety as a trait as determined by STAI X-2 and the QoL of women with the *BRCA1/BRCA2* mutations before and after prophylactic surgery of the reproductive organs (*N* = 61).

The Level of Anxiety as a Trait as Determined by STAI X-2	rho	*p*	rho	*p*
	before RRSO		after RRSO	
Depressed mood (DEP)	−0.54	0.000	−0.49	0.000
Somatic symptoms (SOM)	−0.36	0.004	−0.32	0.013
Memory/concentration (MEM)	−0.43	0.001	−0.39	0.002
Vasomotor symptoms (VAS)	0.09	0.483	−0.10	0.435
Anxiety/fears (ANX)	−0.53	0.000	−0.46	0.000
Sexual behavior (SEX)	−0.30	0.020	−0.23	0.071
Sleep problems (SLE)	−0.41	0.001	−0.47	0.000
Menstrual symptoms (MEN)	−0.30	0.021	−0.18	0.171
Attractiveness (ATT)	−0.50	0.000	−0.37	0.003

RRSO, risk-reducing salpingo-oophorectomy; rho, Spearman’s correlation coefficient; *p*, level of significance.

**Table 5:** The level of stress as determined by the PSS-10 and the QoL of women with the *BRCA1/BRCA2* mutations before and after prophylactic surgery of the reproductive organs (*N* = 61).

Level of Stress as Determined by the PSS-10	rho	*p*	rho	*p*
	before RRSO		after RRSO	
Depressed mood (DEP)	−0.68	0.000	−0.67	0.000
Somatic symptoms (SOM)	−0.39	0.002	−0.36	0.005
Memory/concentration (MEM)	−0.57	0.000	−0.44	0.000
Vasomotor symptoms (VAS)	0.06	0.669	0.00	0.986
Anxiety/fears (ANX)	−0.60	0.000	−0.47	0.000
Sexual behavior (SEX)	−0.34	0.008	−0.26	0.045
Sleep problems (SLE)	−0.49	0.000	−0.46	0.000
Menstrual symptoms (MEN)	−0.35	0.005	−0.41	0.001
Attractiveness (ATT)	−0.46	0.000	−0.36	0.005

RRSO, risk-reducing salpingo-oophorectomy; rho, Spearman’s correlation coefficient; *p*, level of significance.

**Table 6:** The severity of depressive symptoms as determined by the BDI and the QoL of women with the *BRCA1/BRCA2* mutations before and after prophylactic surgery of the reproductive organs (*N* = 61).

Severity of Depressive Symptoms as Determined by the BDI	rho	*p*	rho	*p*
	before RRSO		after RRSO	
Depressed mood (DEP)	−0.69	0.000	−0.72	0.000
Somatic symptoms (SOM)	−0.47	0.000	−0.32	0.013
Memory/concentration (MEM)	−0.60	0.000	−0.52	0.000
Vasomotor symptoms (VAS)	−0.16	0.216	−0.18	0.171
Anxiety/fears (ANX)	−0.63	0.000	−0.56	0.000
Sexual behavior (SEX)	−0.40	0.001	−0.33	0.010
Sleep problems (SLE)	−0.54	0.000	−0.56	0.000
Menstrual symptoms (MEN)	−0.35	0.005	−0.22	0.096
**Attractiveness (ATT)**	−0.59	0.000	−0.36	0.004

RRSO, risk-reducing salpingo-oophorectomy; rho, Spearman’s correlation coefficient; *p*, level of significance.
